# Research progress on the roles of lncRNAs in plant development and stress responses

**DOI:** 10.3389/fpls.2023.1138901

**Published:** 2023-03-07

**Authors:** Xiaoyu Wang, Hai Fan, Baoshan Wang, Fang Yuan

**Affiliations:** Shandong Provincial Key Laboratory of Plant Stress, College of Life Sciences, Shandong Normal University, Ji’nan, Shandong, China

**Keywords:** biological function, characteristics, long non-coding RNA, growth, stress tolerance

## Abstract

Long non-coding RNAs (lncRNAs) are RNAs of more than 200 nucleotides in length that are not (or very rarely) translated into proteins. In eukaryotes, lncRNAs regulate gene expression at the transcriptional, post-transcriptional, and epigenetic levels. lncRNAs are categorized according to their genomic position and molecular mechanism. This review summarized the characteristics and mechanisms of plant lncRNAs involved in vegetative growth, reproduction, and stress responses. Our discussion and model provide a theoretical basis for further studies of lncRNAs in plant breeding.

## Introduction

Long non-coding RNAs (lncRNAs) are RNAs of more than 200 nucleotides (Ponting et al., 2009) that are not translated into proteins or have weak ability to be translated into proteins. lncRNAs were originally thought to be by-products of RNA polymerase II transcription with no biological function (Rinn and Chang, 2012). The importance was overlooked because they have low expression and low sequence conservation and thus considered to be transcriptional “noise”. The first lncRNA X Inactive Specific Transcript (XIST) was discovered in animal cells in 1991 and is involved in the regulation of X chromosome inactivation (Borsani et al., 1991). Thereafter, lncRNAs attracted increased attention in animal research. The first lncRNAs identified in plants were reported in rice (*Oryza sativa* L.) (Kouchi et al., 1999). Subsequently, many lncRNAs have been identified in other plant species, such as Arabidopsis (*Arabidopsis thaliana*), tomato (*Solanum lycopersicum*) (Li et al., 2018), and rice (Kouchi et al., 2010).

Recent research has revealed that lncRNAs have important roles in plant growth and development and their response to abiotic stress. This review discusses the possible roles and regulatory mechanisms of lncRNAs in plant development and stress response, providing a reference for future research on lncRNAs in plants.

### Classification of lncRNAs

**Figure 1:**
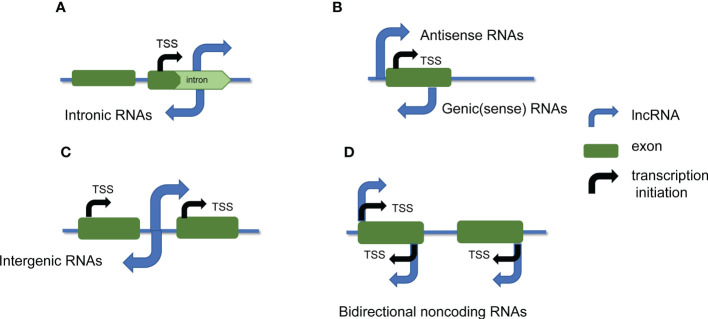
lncRNAs are classified based on their location in the genome. lncRNAs are classified into 5 categories based on their genomic location relative to neighboring protein-coding genes: **(A)** intronic lncRNAs; **(B)** sense lncRNAs and antisense lncRNAs; **(C)** intergenic lncRNAs; **(D)** bidirectional noncoding RNAs. The blue arrows represent lncRNA, the black arrows represent transcription initiation, and the green regions represent exons.

In general, lncRNAs are classified into five types according to their positions in the genome relative to protein-coding genes (**Figure 1**) (Wang and Chekanova, 2017): sense lncRNAs, antisense lncRNAs, bidirectional lncRNAs, intronic lncRNAs, and intergenic lncRNAs, which are also called large interventional ncRNAs or large intervening ncRNAs (lincRNAs)(Fedak et al., 2016; Wu et al., 2020). For sense lncRNAs or antisense lncRNAs, this type is associated with one or more exon phases of another protein-coding gene of the same or opposite strand, respectively overlapped (Pan et al., 2022). The start site of bidirectional lncRNA transcription is very close to the transcription start site encoding the protein gene on the opposite strand, but in the opposite direction. Intronic lncRNA is derived from the intron region of the secondary transcript (sometimes possibly the mRNA precursor sequence). Intergenic lncRNA is produced in the region between two genes.

lncRNAs can also be classified into four categories based on their molecular mechanisms (Wang and Chang, 2011): (1) signaling molecules: lncRNAs that function as signaling molecules to regulate gene expression; (2) bait molecules: lncRNAs that recruit RNA-binding proteins (RBP); (3) guide molecules: lncRNAs that guide RBP complexes to specific target sites (Koziol and Rinn, 2010) or that interact with chromatin remodeling complexes to regulate chromatin changes and to recruit complexes to specific gene loci (Rinn et al., 2007; Nagano and Fraser, 2009; Koziol and Rinn, 2010); (4) skeleton molecules: lncRNAs that act as scaffold molecules by reacting with protein complexes or other effector molecules and participate in the interactions between molecules and influence the accuracy of signaling (Swarbreck et al., 2008; Spitale et al., 2011).

lncRNAs occur in the nucleus and cytoplasm and have similar structural features to mRNAs, such as a 5’ cap, 3’ polytail, and alternative splicing sites (Jonathan et al., 2014), but lncRNAs are generally expressed at lower levels than mRNAs. lncRNA expression varies among tissues, environments, and developmental stages, and lncRNAs can act at the transcriptional, post-transcriptional, or protein levels and be subject to epigenetic regulation. Some lncRNAs play an important role in epigenetic control gene expression by recruiting chromatin reconstitution complexes to specific sites to mediate the expression silencing of relevant genes, such as Xist, Air, HOTAIR, and COLDAIR. lncRNAs are synthesized in the following five ways: (A) protein-coding genes cause the insertion of reading frames, with the inserted reading frame and the previous coding sequence forming a functional lncRNA; (B) chromosome rearrangement connecting two distant non-transcriptional regions, resulting in a lncRNA containing multiple exons; (C) alteration of a non-coding gene to form a functional non-coding inverse gene and a non-functional pseudogene; (D) insertion of a local adjacent two-repeat sequences inside the lncRNA; (E) insertion of a transposable element to form a functional lncRNA (Ponting et al., 2009).

## Methods for studying lncRNAs in plants

Most research on lncRNAs has been conducted in animals or for medical applications, and lncRNA research in plants is relatively lacking. The general strategy for identifying lncRNAs involves (1) high-throughput lncRNA screening and identification; (2) verification of lncRNA expression results obtained from high-throughput analysis; and (3) study of the biological function and mechanism of action of target lncRNAs (Zhu et al., 2013). Common methods for discovering and characterizing lncRNAs include high-throughput sequencing serial analysis of gene expression (SAGE), transcriptome deep sequencing (RNA-seq), cap analysis of gene expression (CAGE), and low-abundance transcript detection/single-cell sequencing (Ma et al., 2012; Kashi et al., 2016). Microarray analysis and RNA-seq are commonly used for high-throughput analysis, and commercialized lncRNA chips are available for humans, rats, mice, and Arabidopsis. After high-throughput analysis, northern imprinting, reverse transcription quantitative PCR (RT-qPCR), and fluorescence *in situ* hybridization (FISH) can be used to verify the lncRNAs. Gain-of-function and loss-of-function studies are used to investigate the biological functions of lncRNAs (Mattick, 2009). Gain-of-function studies involve introducing overexpression vectors, and loss-of-function studies often utilize clustered regularly interspaced short palindromic repeats (CRISPR)/CRISPR-associated protein 9 (Cas9) gene knockout and RNA interference (RNAi). In addition, RNA-pull down experiments, RNA-binding protein immunoprecipitation (RIP), chromatin isolation by RNA purification (ChIRP), capture hybridization analysis of RNA targets (CHART), and crosslinking-immunopurification (CLIP) are used to verify lncRNA targets. *In situ* hybridization (FISH), Fluorescence *in situ* hybridization (FISH) is used for localization studies. RNA and protein interactions have been studied by RNA immunoprecipitation, RIP, high-throughput sequencing cross-linking immunoprecipitation Hits-clip, Photoactivatable ribonucleotide-enhanced cross-linking and immunoprecipitation (PAR-clip); Methods for studying RNA and DNA interaction include Chromatin isolation by RNA purification, ChIRP, RNA Antisense Purification RAP and Capture hybridization analysis of RNA targets (CHART); Methods for studying RNA and RNA interactions include RNA-RAP, Crosslinking, Ligation and sequencing of hybrids, CLASH.

Studies on the mechanisms of action and functional characteristics of lncRNAs have established lncRNA databases for humans and other animals, but few specialized databases exist to host plant lncRNA data. For example, The Arabidopsis Information Resource (TAIR) database is dedicated to Arabidopsis genes and biological model research (Jin et al., 2013). PLncDB (Wang and Chang, 2011) is currently the only published plant lncRNA database that allows convenient lncRNA collection and query. PLncDB contains information on 16,227 Arabidopsis lncRNAs identified through expressed sequence tag (EST) analysis, reproducibility-based tiling array analysis strategy (RepTAS) analysis, chromatin immunoprecipitation (ChIP) analysis, and RNA sequencing (RNA-seq) analysis. In addition, the lncRNAs Database (lncRNAdb)(Quek et al., 2015) contains relevant information such as sequence and structural characteristics, evolutionary conservation, expression, genomic sequence, subcellular localization, functional evidence, and literature links for lncRNAs in plants including Arabidopsis, rice, soybean (*Glycine max*), alfalfa (*Medicago sativa*), grapevine (*Vitis vinifera*), *Brassica rapa*, tomato, and aspen (*Populus tremula*). The Plant Natural Antisense Transcripts DataBase (PlantNATsDB)(Chen et al., 2012) was the first database specifically designed for predicting and querying plant natural antisense transcripts (NATs), which are ncRNAs produced from coding or non-coding antisense sequences and are involved in regulating various biological and abiotic stress-response processes (Lavorgna et al., 2004; Werner and Berdal, 2005; Charon et al., 2010). PlantNATsDB contains approximately 2 million NATs from 69 plant species, including 7,788 NATs (3,005 sense NATs and 4,783 antisense NATs) in Arabidopsis. However, PlantNATsDB only lists NAT pairs and does not allow genome-wide queries.

The CANTATA database (http://yeti.amu.edu.pl/CANTATA/) allows researchers to predict lncRNAs in plants. CANTATA currently contains 239,631 lncRNAs from 39 species and is the largest plant lncRNA database (Quek et al., 2015). NONCODE is a database (http://www.noncode.org/) dedicated to providing the most complete annotation of ncRNAs, especially lncRNAs (Yi et al., 2014). To date, NONCODE contains 548,640 lncRNAs from 17 plant species.

### lncRNAs are involved in vegetative growth

lncRNAs participate in many aspects of vegetative growth, including germination, leaf development, root growth, photomorphogenesis, and phytohormone regulation.

### Leaf development

There are 76 lncRNAs in Arabidopsis(Amor et al., 2009), including 14 antisense transcripts of protein-coding genes that may play a trans-regulatory role; 5 precursors of 24-nt small interfering RNAs (siRNAs); npc83 that are processed by DCL4 to form miR869A; and 22 lncRNAs regulated by abiotic stress. Functional studies indicate that overexpression of npc48 changes miR164 expression and leads to jagged leaves and flowering delay in Arabidopsis, while overexpression of npc536 improves root growth under salt stress. In addition, a rice lncRNA named TWISTED LEAF (TL) maintains flat leaf shape by regulating the expression of an R2R3-MYB transcription factor gene (Liu et al., 2018).

Npc48 was characterized through bioinformatics analysis of a full-length Arabidopsis cDNA library, which identified it as a 983-nt-long lncRNA expressed in roots, stems, leaves, and flowers (Judith Hirsch and PHYSIOLOGY, 2006). Npc48 loss of function results in obvious leaf serrations (a defect of leaf primordial margin cell proliferation) and abnormal vegetative growth, including increased rosette leaf diameter, as well as significantly delayed flowering time. This phenotype is similar to that of the *sucrose ester* (*se*) and *argonaute 1* (*ago1*) mutants. SE regulates miRNA levels and affects miRNA processing. Ago1 is the target cleavage enzyme for most miRNAs, but npc48 overexpression does not affect miRNA or *AGO1* expression, suggesting that it may influence post-transcriptional regulation through other miRNAs (Amor et al., 2009).

### Root growth

Soybean GmENOD40 is a lncRNA originally identified in legumes as associated with the formation of symbiotic nitrogen-fixing nodules (Franssen et al., 1993; van de Sande et al., 1996). Its homologs OsENOD40 and MtENOD40 (Kouchi et al., 2010) were later identified in rice and alfalfa, respectively. OsENOD40 is specifically expressed in rice stems and functions in organ differentiation and vascular tissue development (Kouchi et al., 2010). Yeast three-hybrid experiments found that MtENOD40 in alfalfa interacts with a continuously expressed RNA-binding protein MtRBP1, which shifts MtRBP1 in the cytoplasm, and the subcellular localization change of this RNA-binding protein may represent a new function of lncRNA in cells (Campalans et al., 2004). Yeast three-hybrid experiments also revealed that MtENOD40 interacts with two nodulin acid RNA-binding proteins, small nodulin acidic RNA-binding proteins 1 and 2 (MtSNARP1 and MtSNARP2)(Laporte et al., 2010). These studies show that the yeast three-hybrid assay technique can be used to screen biological macromolecules that interact with lncRNAs.

The β-glucuronidase (GUS) expression pattern of *ENOD40-GUS* transgenic plants suggests that tomato ENOD40 may be associated with the elimination of ethylene (Vleghels et al., 2003). Further, in soybean, ENOD40 encodes 12- and 24-amino-acid short peptides that specifically bind to sucrose synthases, indicating that ENOD40 is associated with the regulation of sucrose utilization in nodules. Alfalfa ENOD40 encodes short peptides of 13 and 27 amino acids, which are associated with ENOD40 biological activity (Sousa et al., 2001; Rohrig et al., 2002). However, the mechanism of action of ENOD40 may occur *via* ENOD40’s RNA molecule rather than its encoded peptides (Zhu and Wang, 2012)

Two Arabidopsis nuclear speckle RNA binding proteins (NSRs), NSRa and NSRb, are required for selective and/or constitutive splicing (Schindler et al., 2008). A lncRNA called Alternative Splicing Competitor lncRNA (ASCOlncRNA, formerly known as Npc351) competes with precursor mRNAs for binding to AtNSRs. ASCOlncRNA-overexpression lines accumulate alternatively spliced transcripts. Auxin treatment induces NSRb accumulation and increases lateral root formation in wild-type seedlings, but double mutants of NSRa and NSRb produce very few lateral roots and are insensitive to auxin-induced lateral root formation. Thus, ASCOlncRNA regulates alternative splicing during root development by binding to AtNSRs after perceiving auxin signals (Bardou et al., 2014).

Nitrogen is critical for plant growth and development, and improving the nitrogen utilization efficiency of crops for sustainable agriculture is currently a major goal in plant breeding (Liu et al., 2019). Six nitrate-induced lncRNAs were identified by combining RNA-seq and RT-qPCR, and the function of the lncRNA most strongly induced by nitrate, T5120, was explored in depth. The expression of a nitrate-responsive gene was significantly increased in a T5120-overexpression line, indicating that T5120 regulates the plant’s response to nitrate. Furthermore, the NIN-LIKE PROTEIN 7 (NLP7) transcription factor, involved in nitrate signaling, binds to the T5120 promoter and regulates T5120 expression, and T5120 acts on the downstream regulation of nitrate signaling of NLP7. Nitrate reductase activity and amino acid content were significantly elevated in the T5120-overexpression line, indicating that T5120 enhances nitrate assimilation in plants. In addition, T5120 over expression increased plant biomass and promoted root development, thereby improving plant growth.

### Photomorphogenesis

In Arabidopsis *PHYTOCHROME INTERACTING FACTOR 3* (*PIF3*) encodes a basic helix-loop-helix (bHLH) transcription factor that inhibits photomorphogenesis. In Arabidopsis the lncRNA HIDDEN TREASURE 1 (HID1) negatively regulates *PIF3*, thereby promoting photomorphogenesis in seedlings (Wang et al., 2014). Knockout of *HID1* increases *PIF3* transcription, resulting in significantly longer hypocotyls in the knockout mutant compared to wild-type seedlings (Kim et al., 2003).

### Phytohormone regulation

**Figure 2:**
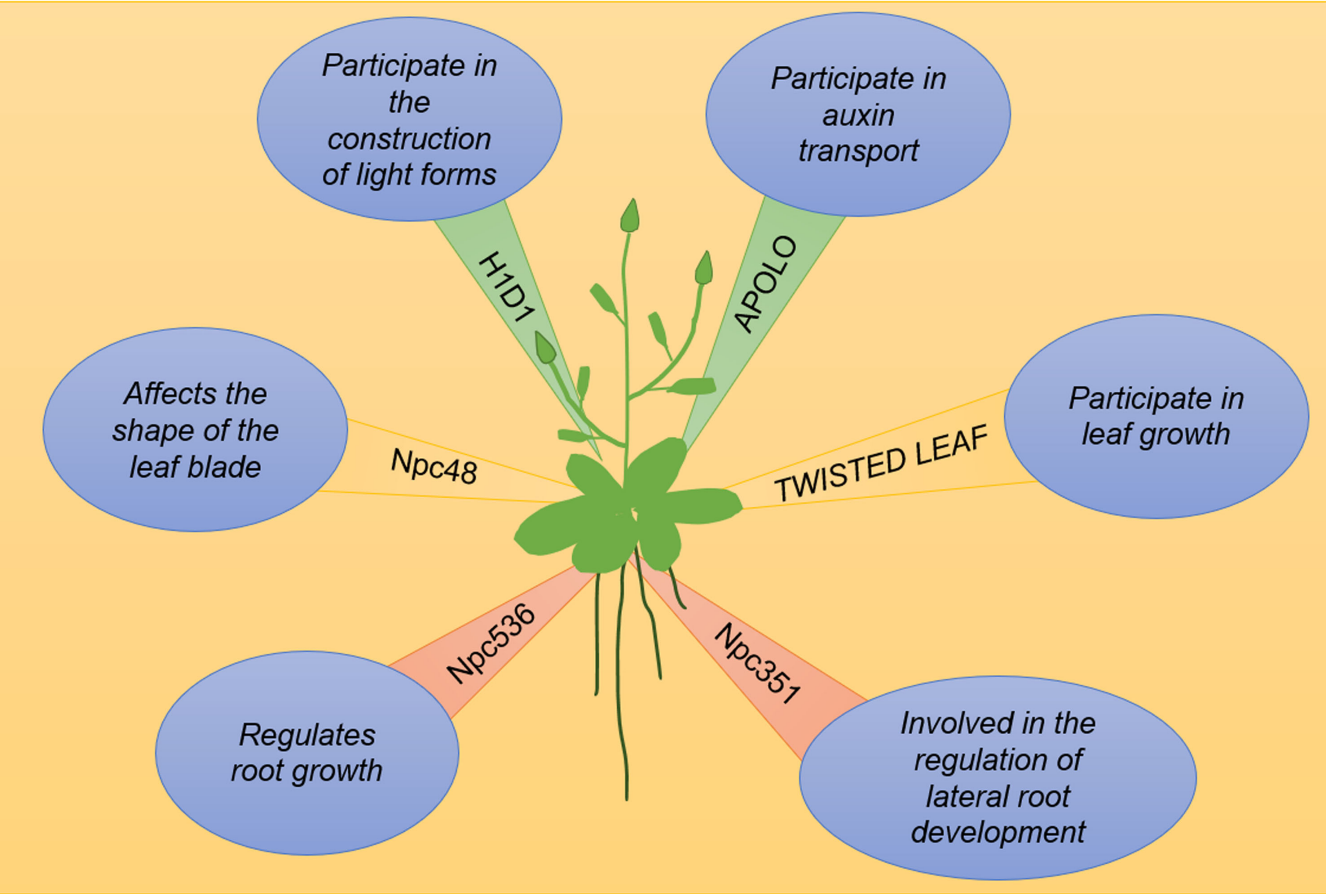
lncRNAs are involved in plant growth and development. H1D1 inhibits photomorphogenesis, APOLO is the promoter ring regulated by auxin, TWISTED LEAF maintains the flattening of leaves, Npc48-overexpressing plants show obvious leaf serrations, npc536 overexpression regulates Arabidopsis root growth, and Npc351 is an alternative splicing competitor.

lncRNAs are involved in auxin transport and signaling. A 5-kb-long lncRNA transcribed by RNA polymerase II and RNA polymerase V, named auxin-regulated promoter loop (APOLO), is a key regulator in auxin-polar transport. APOLO dynamically regulates the formation of chromatin ringsof the downstream gene *PINOID* (*PID*). Upon treatment with exogenous auxin, the APOLO site undergoes demethylation and the chromatin ring is opened to facilitate binding to transcription factors (Ariel et al., 2014). **Figure 2** illustrates a working model of the role of lncRNAs in vegetative growth. The lncRNAs known to be involved in vegetative growth are listed in **Table 1**.

**Table 1:** lncRNAs involved in vegetative growth.

lncRNA name	Species	Biological function	References
**Npc48**	Arabidopsis	Affects leaf blade shape	(Hirsch et al., 2006)
**Npc536**	Arabidopsis	Regulates root growth	(Hirsch et al., 2006)
**Npc351**	Arabidopsis	Involved in the regulation of lateral root development	(Schindler et al., 2008)
**T5120**	Arabidopsis	Promotes root development	(Liu et al., 2019)
**HID1**	Arabidopsis	Participates in the construction of light forms	(Wang et al., 2014)
**APOLO**	Arabidopsis	Participates in auxin transport	(Ariel et al., 2014)
**ASCO−RNA**	Arabidopsis	Involved in auxin transport and developmental signal output regulation	(Bardou et al., 2014)
**GmENOD40**	Legumes	Involved in the formation of nitrogen-fixing nodules in legumes	(Franssen et al., 1993; van de Sande et al., 1996)
**MtENOD40**	Lucerne	Alters subcellular localization	(Kouchi et al., 2010)
**TWISTED LEAF**	Rice	Participates in leaf growth	(Liu et al., 2018)
**OsENOD40**	Rice	Affects organ differentiation and vascular tissue development	(Kouchi et al., 2010)
**ENOD40**	Tomato	Eliminates ethylene	(Zhu and Wang, 2012)

### lncRNAs are involved in reproduction

**Figure 3:**
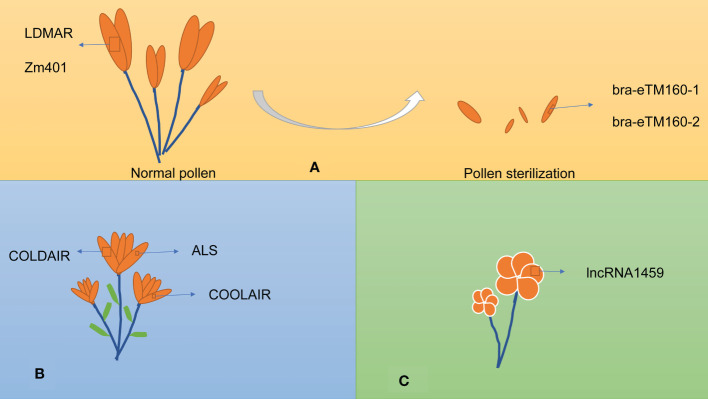
lncRNAs influence various aspects of plant reproduction. **(A)** lncRNAs involved in pollen development. **(B)** lncRNAs involved in vernalization. **(C)** lncRNAs involved in fruit ripening.

lncRNAs are also involved in reproduction, including pollen development, flowering time, and fruit ripening. The lncRNAs involved in reproduction are listed in **Table 2** and exemplified in **Figure 3**.

**Table 2:** lncRNAs participating in reproduction.

lncRNA name	Species	Biological function	References
**COLDAIR**	Arabidopsis	Participates in the vernalization response to induce flowering	(Yamaguchi and Abe, 2012; Bardou et al., 2014)
**COOLAIR**	Arabidopsis	Participates in the vernalization response to induce flowering	(Yamaguchi and Abe, 2012; Bardou et al., 2014)
**ALS**	Arabidopsis	Participates in the vernalization response to induce flowering	(Shin and Chekanova, 2014)
**MAS**	Arabidopsis	Regulates flowering time	(Yang et al., 2022)
**Zm401**	Corn	Regulates pollen development	(Ariel et al., 2014)
**LDMAR**	Rice	Regulates pollen development and photosensitive male infertility	(Ding et al., 2012)
**bra-eTM160-1**	Turnip	Participates in pollen development	(Huang et al., 2018)
**bra-eTM160-2**	Turnip	Participates in pollen development	(Huang et al., 2018)
**lncRNA1459**	Tomato	Participates in fruit ripening	(Li et al., 2018)
**MdLNC499**	Apple	Participates in fruit ripening	(Ma et al., 2021).

### Pollen development

A rice lncRNA called long-day-specific male-fertility-associated RNA (LDMAR) is associated with photosensitive male infertility; normal pollen development requires a sufficient amount of LDMAR transcripts. A single-nucleotide polymorphism (SNP) results in increased methylation of the *LDMAR* promoter, which reduces its transcription. Lack of LDMAR results in premature programmed cell death of anthers under long days, eventually leading to photosensitive male infertility (Ding et al., 2012).

It is worth to mention that RdDM is also an important regulatory model in plant development, such as lncRNA LDMAR and lncRNA identified from sex determination in Populus. The small telomere peri-telomere X and Y-linked regions of *Pseudomonas trigonum* chromosome XIX are sequenced. Both genes are present only in the Y-linked region. One of them is a member of the LTR/Gypsy transposable element family, which produces long noncoding RNA, and the overexpression of this gene in Arabidopsis promotes androgen development (Xue et al., 2020).

A 1149-nt-long lncRNA in maize (*Zea mays*), Zm401, regulates the expression of three key genes related to pollen development: ZmMADS2, MZm3-3, and ZmC5. Zm401 inhibition or overexpression affects the growth and development of stamens and anthers, and eventually causes microspores to empty and deform. In Zm401-deletion mutants, MZm3-3 is upregulated, while ZmMADS2 and ZmC5 are down-regulated, resulting in abnormal microspore and velvet layer development, ultimately causing male infertility. Zm401 overexpression causes anther degradation, negatively affecting pollen development (Ariel et al., 2014).

LncRNA acts as an endogenous microRNA target mimic (eTM) to regulate miRNA expression and function. During pollen development in turnip (*Brassica rapa* subsp. *rapa*), lncRNAs function as targets to regulate the function of miRNAs (eTMs), of which bra-eTM160-1 and bra-eTM160-2, are predicted to be eTMs of bra-mir160-5p. By overexpressing the eTMs in *B. rapa*, bra-eTM160-1 and bra-eTM160-2 had inhibitory ability on bra-miR160-5p and participated in pollen formation and male fertility through upregulating ARF genes, especially BrARF17. Although transgenic plants have a normal flower shape, half of the pollen grains in the anthers are small and wrinkled, are non-viable, and lack the nucleus and pollen lining (Huang et al., 2018).

Homozygous tetraploid rice has advantages over diploid rice in terms of genetic evolution, but these are often accompanied by low fertility and poor seed set. High-throughput sequencing revealed that the lncRNAs associated with transposable elements and meiosis may be endogenous regulators of pollen and blastocyst development, and their differential expression leads to decreased fertility in homozygous tetraploid rice (Li et al., 2020).

To investigate the role of lncRNAs in cotton (*Gossypium hirsutum*) anthers, Zhang et al. performed transcriptome sequencing during anther development in trilineage hybrid cotton (cytoplasmic male sterility line, holding line, and recovery line), and identified 80,695 candidate lncRNAs involved in cytoplasmic male infertility and fertility restoration. A subsequent gene ontology (GO) analysis of the lncRNAs that were differentially expressed in sterile lines (the male organs of the plant that degenerate or develop abnormally, lose the ability to fertilize, and are not strong in self-breeding, but the pistils are normal and can accept normal pollen from other plant varieties that can bear strains) vs. retention lines (the male sterility preservation line) and in sterile lines vs. recovery lines (male sterility recovery line) showed that the sterile lines - retention lines were metabolized in cytohormonals. They found significant differences between the cytokinesis process and the redox reaction process, but the main difference between the sterile line and the recovery line was in the genes regulating cell morphology during fertility recovery (Zhang et al., 2019). Analysis of the differences in the expression of lncRNAs and mRNAs in the pollen mother cell stage, the tetrachosic stage, and the small spore stage between the sterile line and the holding line revealed that these lncRNA and mRNAs may be involved in reproductive development processes, such as velvet layer cell degradation, microspore development, pollen development, and the differentiation, proliferation, and apoptosis of anther cell walls. GO and Kyoto Encyclopedia of Genes and Genomes (KEGG) analysis revealed their involvement in plant oxidative phosphorylation and flavonoid biosynthesis, pentose and gluconic acid are converted to each other, fatty acid biosynthesis, and map signaling pathways lead to anther failure due to abnormal metabolic pathways, ultimately resulting in male infertility (Li Y. et al., 2019).

### Flowering induction

The lncRNAs COOLAIR and COLDAIR are associated with Arabidopsis *FLOWERING LOCUS C* (*FLC*), a flowering inhibitor. COOLAIR is a NAT of *FLC*, and COLDAIR is transcribed from the first intron of *FLC*. COOLAIR and COLDAIR bind to the polycomb repressive complex 2 (PRC2) protein complex, causing *FLC* chromatin histone methylation and ultimately promoting flowering (Bardou et al., 2014). COOLAIR expression rises sharply in the early spring. Under a low-temperature vernalization treatment of about 20 days to induce flowering, COLDAIR expression was initially low, increased throughout the treatment, and peaked at 20 days of vernalization (Yamaguchi and Abe, 2012).

Inhibition of Flowering Site C (FLC) expression is a key switch for regulating flowering. Two coding nuclear exosome components in Arabidopsis AtRRP6L1 and AtRRP6L2 gene mutations lead to desuppression of the major flowering inhibitor FLC, thereby delaying the flowering of the early-flowering ecotype (Shin and Chekanova, 2014). Interesting, a novel, long non-coding, non-polyadenylated antisense transcript (ASL, for Antisense Long) was originated from the FLC locus. Different RNAs (AtRRP6L1 and AtRRP6L2) generated from FLC sites may have different functions in altering the epigenetic environment of FLC sites, and play a role in regulating these noncoding RNAs.

Arabidopsis encodes a NAT lncRNA named MAS, a key component of the histone-binding methyl R transferase WDR5a (WD40 containing repeat 5a). MAS is recruited to the *MADS AFFECTING FLOWERING4* (*MAF4*) locus, where it enhances histone 3 lysine 4 trimethylation (H3K4me3) to activate *MAF4* expression (Zhao et al., 2018), thereby regulating the flowering time.

### Fruit ripening

A lncRNA1459 deletion mutant was obtained by CRISPR/Cas9 gene editing to study its function in tomato fruit ripening (Li et al., 2018). Compared with the wild type, tomato lncRNA1459-knockout mutants showed significant inhibition of fruit maturation, ethylene synthesis, and tomato red accumulation. Further, the expression of many fruit ripening genes and lncRNAs also changed significantly: 81 upregulated DELs and 31 downregulated DELs have been discovered with repression of lncRNA1459, indicating that lncRNA1459 regulates gene and lncRNA transcription during tomato fruit ripening.

In apple, photo-induced lncRNA MdLNC499 was involved in anthocyanin accumulation (Ma et al., 2021). MdLNC499 overexpression or silence in apple fruit and calluses showed that both anthocyanin accumulation and transcription levels of MdERF109 were dependent on MdLNC499, indicating that MdLNC499 positively regulates the expression of MdERF109 to promote light-induced anthocyanin biosynthesis.

### The role of lncRNAs in abiotic stress

**Figure 4:**
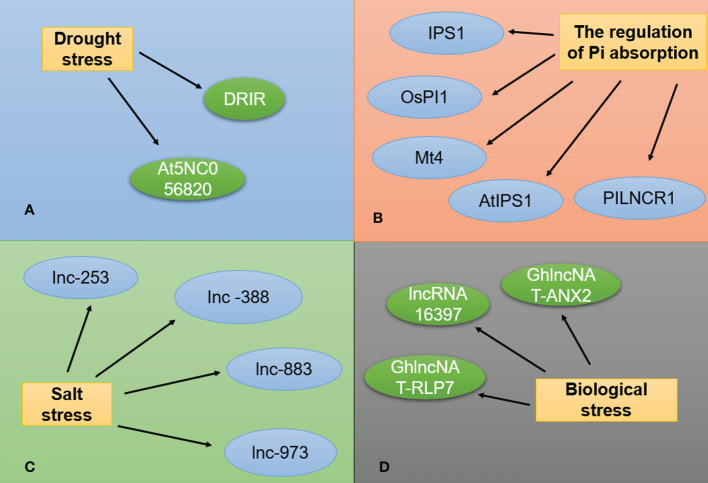
lncRNAs are involved in stress responses. **(A)** Two Arabidopsis lncRNAs associated with drought stress. **(B)** lncRNAs associated with phosphorus stress. **(C)** lncRNAs associated with salt stress. **(D)** lncRNAs associated with biotic stress.

In addition to participating in all developmental stages of plants, lncRNAs also play important roles in the response to abiotic stresses, such as low temperature, drought, and salinity (**Figure 4**). The lncRNAs known to be involved in abiotic stress response are listed in **Table 3**.

**Table 3:** lncRNAs involved in abiotic stress responses.

lncRNA name	Species	Biological function	References
**DRIR**	Arabidopsis	Responds to drought stress	(Qin et al., 2017)
**At5NC056820**	Arabidopsis	Responds to drought stress	(Liu et al., 2012; Ruonan et al., 2017)
**AtIPS1**	Arabidopsis	Regulates the absorption of Pi	(Jian et al., 2013)
**At4**	Arabidopsis	Regulates the absorption of Pi	(Franco-Zorrilla et al., 2007)
**lnc_388**	Cotton	Responds to salt stress	(Ma et al., 2008)
**lnc_883**	Cotton	Responds to salt stress	(Ma et al., 2008)
**lnc_973**	Cotton	Responds to salt stress	(Ma et al., 2008)
**lnc_253**	Cotton	Responds to salt stress	(Ma et al., 2008)
**Mt4**	Lucerne	Regulates the absorption of Pi	(Burleigh and Harrison, 1997; Burleigh and Harrison, 1998; Burleigh and Harrison, 1999)
**TPSI1**	Tomato	Regulates the absorption of Pi	(Mendu, 2010)
**PHO1;2**	Maize	Regulates the absorption of Pi	(Franco-Zorrilla et al., 2007; Du et al., 2018)

### Drought stress response

Under normal conditions, the expression of Arabidopsis drought-induced lncRNA (*DRIR*) is low, but its expression increases significantly under droughtstress, and Arabidopsis plants overexpressing *DRIR* are more tolerant of drought stress than wild-type plants (Qin et al., 2017). The lncRNA *At5NC056820* was identified in Arabidopsis under drought stress and abscisic acid (ABA) treatment. *At5NC056820*-overexpressing Arabidopsis lines are more tolerant of drought than wild-type plants (Liu et al., 2012; Ruonan et al., 2017).

Transcriptome analysis of drought-tolerant rapeseed (*Brassica napus*) and drought-sensitive Brassicales under drought and rehydration conditions was used to construct a lncRNA-mRNA co-expression network, which identified lncRNAs involved in phytohormone signal transduction during drought, and verified that these lncRNAs positively regulate the expression of target genes to respond to drought stress (Tan et al., 2020).

Researchers (Li et al., 2019) used whole-transcriptome strand-specific RNA sequencing to obtain candidate differentially expressed genes associated with rice drought “memory”. This study demonstrated that rice forms a drought “memory” under suitable repeated drought treatments and explored the underlying mechanism. The results showed that lncRNAs and phytohormones (especially ABA) are involved in the formation of this short-term drought “memory” and activate the expression of “memory” transcripts in metabolic pathways such as photosynthesis and proline synthesis, thereby improving the plant’s ability to cope with drought stress.

### Salt stress response

A transcriptome analysis of trifoliate cotton seedlings identified 1,117 unique lncRNAs expressed under salt stress, and reported that lnc_388 may regulate gh-A09G1182 MS; lnc_883 improved salt stress tolerance by adjusting the GH-D03G0339 MS channel; and lnc_973 and lnc_253 regulate ghr-miR399 and ghr-156e under salt stress (Ma et al., 2008).

Transcriptome analysis of the salt-tolerant maize ‘Lluteño’ (Huanca-Mamani et al., 2018) after salt stress treatment identified 48,345 lncRNAs, of which 41.9% belonged to ‘Lluteño’ maize. This group identified and verified the expression patterns of antisense transcripts that may affect transcriptional regulation, stress responses, responses to abiotic stimuli, and genes involved in nicotinic metabolic processes.

A transcriptome analysis of alfalfa lncRNAs under salt stress (Wang et al., 2015) found that the number of lncRNAs differentially expressed under salt stress was higher in roots than in leaves, while the opposite pattern was observed in response to osmotic stress. Transcriptome sequencing of the top buds of *Paulownia tomentosa* seedlings (Wang et al., 2018) treated with the mutagen methyl methane sulfonate (MMS) identified 2,531 candidate lncRNAs. Among these lncRNAs, seven were identified as precursors of 13 known small RNAs, and 15 may be used as targeted simulations of 19 small RNAs. The authors identified 220 lncRNAs responding to MMS, including 7 phytohormone-associated lncRNAs, and 1 lncRNA involved in base excision repair. lncRNAs are involved in many biological processes in plants (Nejat et al., 2017), but systematic studies of post-transcriptional regulation by lncRNAs in stress responses are lacking.

Transcriptome sequencing (Sun et al., 2018) following cold, heat, drought, and salt stress in rice yielded over 7,000 lncRNAs, nearly half of which were newly discovered. Most of the approximately 500 polyadenylated lncRNAs differentially expressed under stress were significantly downregulated. In addition, hundreds of downregulated polyadenylated lncRNAs were highly conserved and co-expressed with stress-induced protein-coding genes. Many downregulated polyadenylated lncRNAs have been observed in arid and saline rice, suggesting that lncRNA polyadenylation and subcellular localization may be regulated at the post-transcriptional level.

### Cd stress response

Recently, lncRNA was found to play a role in Cd stress response (Quan et al., 2020). Detected using insertions/deletions, two Cd-responsive lncRNA genes of Populus, MSTRG.22608.1–PtoMYB73 and MSTRG.5634.1–PtoMYB27, were identified as polymorphisms driving target gene expression. Genotype analysis of lncRNAs and heterologous overexpression of PtoMYB73 and PtoMYB27 in Arabidopsis indicated their effects on enhancing Cd tolerance, photosynthetic rate, and leaf growth, and the potential interaction mechanisms of PtoMYB73 with abiotic stresses.

### Regulation of inorganic phosphate (Pi) absorption

*Tomato phosphate starvation induced 1* (*TPSI1*) is a well-studied stress-response gene encoding a lncRNA in tomato, and the *TPSI1* gene family is expressed under phosphorus stress. *TPSI1* homologs were also identified in Arabidopsis (*INDUCED BY PHOSPHATE STARVATION1* (*IPS1*)) and maize (*Pi-deficiency-induced long-noncoding RNA1* (*PILNCR1*)), and found to compete with miR399 to bind to PHOSPHATE2 (PHO2), thereby enhancing PHO2 accumulation to maintain normal growth under phosphorus stress (Franco-Zorrilla et al., 2007).

The alfalfa *Mt4* (Burleigh and Harrison, 1997; Burleigh and Harrison, 1998; Burleigh and Harrison, 1999) sequence contains several short open reading frames, and a partial sequence of one of these coincides with the open reading frame of *TPSI1* in tomato. Under phosphate starvation, *Mt4* is strongly induced in alfalfa roots, but under phosphate-sufficient conditions, *Mt4* is barely transcribed.

Another member of the *TPS11*/*Mt4* family, Arabidopsis *INDUCED BY PHOSPHATE STARVATION1* (*IPS1*), accumulates in leaves and roots under phosphate-starvation conditions (Mendu, 2010). Arabidopsis miR399, which is also induced by phosphate starvation, accumulates in the aboveground parts and roots and mediates *PHO2* mRNA cleavage through complementary base pairing in the 5′untranslated region of *PHO2*, reducing *PHO2* transcript levels (Fujii et al., 2005; Franco-Zorrilla et al., 2007). The 23-nucleotide conserved regions in *AtIPS1* are also complementary to miR399 with central mismatches. *AtIPS1* inhibits miR399 activity by competing for binding to *PHO2*, which increases *PHO2* transcript levels to maintain normal growth under phosphate deficiency (Mendu, 2010; Jian et al., 2013). Therefore, *AtIPS1* inhibits miRNA activity through a “target mimicry” mechanism (Jian et al., 2013), and is the first member of the *TPSI1/Mt4* family for which a definitive mechanism has been reported. The mechanism of action of *AtIPS1* indicates that some lncRNAs pair with functional miRNAs through partial base pairing; therefore, miRNAs can imitate target genes, weakening the regulatory role of miRNAs, and playing an important role in biological processes. This provides a strategy for predicting lncRNA target elements with unknown functions.

Another member of the *TPSI1*/*Mt4* family in Arabidopsis, the 747-nt *At4*, localizes to roots and stems and is induced under phosphorus stress. *At4* loss of function inhibits phosphorus distribution to the roots under phosphorus stress, while phosphorus accumulates in the stem. Conversely, *At4* overexpression decreases the phosphorus content in roots (Franco-Zorrilla et al., 2007; Shin et al., 2010). Transgenic plants overexpressing *AT4*, *IPS1*, or both exhibited the same phosphorus content phenotypes (Franco-Zorrilla et al., 2007), indicating that At4 and IPS1 are functionally redundant under phosphorus stress.

Rice *PHOSPHATE1;2* (*PHO1;2*) encodes a protein responsible for loading phosphoric acid into the xylem. The *PHO1;2* complementary strand encodes a related cis-lncNAT. *PHO1;2* and the cis-lncNAT are controlled by active promoters and expressed in vascular tissues, but phosphorus starvation is induced only by the cis-lncNAT promoter. Under phosphorus stress, *PHO1;2* and cis-lncNAT accumulate, but *PHO1;2* mRNA levels remain stable. Down-regulating cis-lncNAT expression through RNAi decreases *PHO1;2* levels, disrupts phosphorus transport from the rhizome to the stem, and reduces seed yield, while constitutive overexpression of cis-lncNAT strongly increases *PHO1;2*, even under phosphorus deficiency. Cis-lncNAT expression is associated with transport of justice-antonym pairs to polysomes, suggesting that cis-lncNAT facilitates *PHO1;2* translation and affects phosphorus dynamics (Jabnoune et al., 2013).

### lncRNAs involved in biotic stress responses

lncRNAs are also involved in bacterial, fungal, and other biological stresses. Comparing tomato plants that are resistant and susceptible to *Phytophthora* (the causal agent of tomato late blight) revealed that *lncRNA16397* induces *SlGRX22* expression and increases tomato resistance to late blight. Whole-transcriptome bioinformatics analysis in Arabidopsis revealed that a 983-nt lncRNA (*lncRNA-Npc48*) is expressed in roots, stems, leaves, flowers, and other tissues. Cotton plants with silenced lncRNAs (GhlncNAT-ANX2 and GhlncNAT-RLP7) had increased resistance to the fungal phytopathogens *Verticillium dahliae* and *Botrytis cinerea*, possibly associated with enhanced *LOX1* and *LOX2* expression (Zhang et al., 2015).

Plants overexpressing npc48 show leaf deformities (Amor et al., 2009). A lncRNA in cucumber (*Cucumis sativus*) called *CsM10* (for *C. sativus* male-specific clone 10), located in the telomere region of chromosome 6, is expressed in varying amounts in different tissues. Three noncoding RNAs associated with biological stress (GenBank Accession Nos. BX827695, D79216, and BX819089) have conserved sequences within the same 179-bp region of each gene, indicating that they may have similar biological stress-response functions. This potential functional conservation could be validated by observing the phenotypes of overexpression and knockout plants of these genes at various stages (Cho et al., 2006).

Moreover, different from the conventional lncRNA structures and functions, some lncRNAs also had the capacity to encode open reading frames (sORFs) which also play important roles in plant development and responses. SORFs-encoded short peptides (SEPs) have been demonstrated to play a crucial role in regulating the biological processes such as growth, development, and resistance response (Zhao et al., 2021). Additionally, more and more important molecular mechanisms of lncRNA for interested traits are constantly discovered.

## Perspective

High-throughput sequencing technologies and bioinformatics have accelerated the discovery of lncRNAs. While progress has been made in dissecting the molecular mechanisms, functions, and characteristics of lncRNAs, many lncRNAs remain to be discovered and characterized. Compared to the robust lncRNA research in animals, lncRNA research in plants is still in its infancy. Plant lncRNAs are known to be involved in vernalization, pollen development, stress responses, and many other biological processes, studies on the molecular mechanisms of lncRNAs and their roles in transcriptional regulation and plant growth and development are lacking as compared to those in animals, and methods for studying lncRNAs in animals need to be extrapolated to plant lncRNAs.

The main problems are: (1) lack of lncRNA gene chips (biochips are currently available only for Arabidopsis among plant species), (2) imperfect genome and protein databases, (3) limited knowledge on the functions and mechanisms of lncRNAs in plants, and (4) scarcity of new technologies and resources for lncRNA research. lncRNA performs co-expression analysis and transcription factor prediction of target genes and differential mRNA, and is applied in plant genetic engineering by CRISPR/Cas9 gene editing technology.

The following advances are needed to facilitate lncRNA research in plants: (1) improve the plant genome and proteome databases; (2) develop a new generation of transcriptome sequencing technology; (3) strengthen the application of bioinformatics theories and methods for discovery, expression, and functional prediction of plant lncRNAs; and (4) design lncRNA research methods according to the characteristics of plant growth and development and cell composition. Our current understanding of plant lncRNAs represents just the tip of the iceberg. We have transitioned from screening lncRNAs to annotating their functions and investigating their mechanisms of action. In addition, for the data information of the structural characteristics and mechanism of action of lncRNA, the establishment of a new research technology system will be an important research direction in the future.

## Author contributions

Conceptualization, FY. Writing – Original Draft, XW and HF. Writing – Review & Editing, FY and BW. All authors contributed to the article and approved the submitted version.
